# Efficacy of Psychological Interventions for Patients with Breast Hyperplasia

**DOI:** 10.1007/s12013-014-0388-4

**Published:** 2015-01-30

**Authors:** Cxian-chun Chen, Cheng-gang Jiang, Qing-qiu Chen, Dong Gao

**Affiliations:** Department of Sleep and Psychology, Institute of Surgery Research, Daping Hospital, Third Military Medical University, Chongqing, 400042 China

**Keywords:** Breast hyperplasia, Psychological intervention, Efficacy evaluation

## Abstract

To explore the efficacy of psychological interventions (PI) in patients with breast hyperplasia (BH). In total 120 BH patients who were treated in the Third Affiliated Hospital of the Third Military Medical University were randomly divided into PI group (*n* = 40; treated with oral XiaoYao Pill and psychological interventions), anti-anxiety/depression medication (AADM) group (*n* = 40; treated with oral XiaoYao Pill and paroxetine), and control group (*n* = 40; treated with oral XiaoYao Pill) and the treatment lasted for 1 year. Before the treatment and 4, 8, and 12 months after the initiation of treatment, the changes in the psychological indicators were measured using Toronto Alexithymia Scale (TAS), Hamilton Anxiety Scale (HAMA), Hamilton Depression Scale (HAMD), and Ways of Coping Questionnaire (WCQ), as well as the physiological indicators including estradiol, prolactin, and progesterone were determined. The overall response rates were evaluated at the end of the treatment, and the relapse rates were calculated during the 1-year follow-up. The HAMD and HAMA scores were declined in all three groups. The scores of TAS and WCQ negative coping subscales showed a declining trend after treatment for the AADM and PI groups. Compared to the control and PI groups, the HAMA and HAMD scores were significantly lower in the AADM group 4, 8, and 12 months after the initiation of treatment (*P* < 0.05). The scores of TAS and WCQ negative coping subscales were significantly lower in AADM group but were significantly higher than those in PI group and lower than the control group 4, 8, and 12 months after the initiation of treatment (*P* < 0.05). The HAMA and HAMD scores were significantly lower in PI group than in control group 4, 8, and 12 months after the initiation of treatment (*P* < 0.05). After the initiation of treatment, the estradiol and prolactin levels decreased while the progesterone levels increased in all three groups. Compared with the control group and AADM group, the PI group had significantly higher estradiol and prolactin levels and higher progesterone levels 4, 8, and 12 months after the initiation of treatment (*P* < 0.05). Compared with the control group, the AADM group had significantly lower levels of estradiol and prolactin and higher progesterone levels 4, 8, and 12 months after the initiation of treatment (*P* < 0.05). The overall response rate was not significantly different for both PI group and AADM group (*P* > 0.05), while the relapse rate was significantly lower in the PI group than the control and AADM groups (*P* < 0.05). However, the relapse rate did not significantly differ between the control group and AADM group (*P* > 0.05). PI can effectively improve the psychological status of BH patients and restore the disordered endocrine system. The efficacy lasts for long and the relapse rate is lower. Therefore, it could be an effective method for treating BH.

## Introduction

Breast hyperplasia (BH) is one of the leading breast disorder, and its prevalence is increasing annually, therefore posing severe threats to the physical/mental health and quality of life of women [1–3]. BH can be caused by a variety of factors. In addition to biological factors, the role of psychological factors in the pathogenesis of BH has increasingly been recognized. The psychological factors are related with human nervous system, immunity, and endocrine system, therefore can directly or indirectly affect the development and progression of BH [4]. In our current study, we applied the psychological interventions (PI) in treating patients with BH.

## Subjects and Methods

### Subjects

A total of 120 BH patients who were treated in our department from January 2010 to June 2010 were assigned into control group (*n* = 40), anti-anxiety/depression medication (AADM) group (*n* = 40), and psychological intervention (PI) group (*n* = 40) using a random digit table. The inclusion criteria: a) meet the diagnostic criteria of BH described in the *Diagnosis and Treatment of Breast Diseases* (2nd Edition) [5]; and b) with a Hamilton Anxiety Scale (HAMA) score of >14 and a Hamilton Depression Scale (HAMD) score of >17. The exclusion criteria included: (a) having other breast lesions in addition to BH; (b) use of steroids within the past one month; (c) with a family history of breast cancer; (d) with atypical mass in the breast, which was not responsive to previous drug therapies; and (e) with an education background lower than middle school and/or could not understand the content of scales. All the patients in the treatment groups signed the informed consents and the study was approved by the Hospital Ethics Committee. The age, disease location, years of education, and disease course showed no significant differences among the three groups (*P* > 0.05) (Table 1).

**Table 1:** General condition of patients in all three groups

Group	Site of lesion (unilateral/bilateral)	Age (years)	Years of education (years)	Disease course (months)
PI group	15/25	39.12 ± 11.78	12.36 ± 1.67	84.91 ± 23.25
AADM group	10/30	37.61 ± 13.53	12.15 ± 1.90	80.35 ± 21.56
Control group	12/28	36.54 ± 11.56	12.67 ± 0.98	81.15 ± 21.35
*F*/χ^2^ value	1.485	0.328	0.213	0.827
*P* value	0.476	0.643	0.821	0.510

### Methods

#### Treatment

Patients in control group were treated with oral Xiaoyao Pill (Wanxi Pharmaceutical Co. Ltd, Henan, China; licensing number: Z41021831) 3–9 g, tid, for one year. Patients in AADM group were treated with oral Xiaoyao Pill and paroxetine (Seroxat^®^, Smith Kline Pharmaceutical Co., Ltd., Tianjin, China; licensing number: H10950043) 10–20 g, qd, for one year. Patients in PI group were given oral Xiaoyao Pill and PI for one year. The integrated PI included the following three aspects: (1) cognitive-behavioral therapy: once weekly for 40–60 min, during which the knowledge of breast structure, proper breast hygiene, mechanisms of proliferative breast diseases, and routine precautions of breast diseases were explained to the patients. (2) Systemic family therapies, which included therapeutic interviews (twice weekly for 60–90 min using techniques including circular questioning, difference-making questioning, feed-forward questioning, feedback questioning, de-diagnosing) and homework assignments (including homework for odd-numbered and even-numbered days, keeping secret progress account, role reversal, and life-changing homework assignments). (3) Group psychotherapy: Closed structured groups (8–12 persons in each group) were set for the group psychotherapy (once weekly for 90–120 min), and included following four stages: (a) initial phase: the group members became familiar with each other to establish trust relationship, and group-related policies were established; (b) transition phase: the group members communicated deeply about their diseases, became empathized, and established a sense of belonging; (c) working phase: the group members further expressed their true feelings about the disease, and they got feedback from each other and encouraged such discussion, so as to establish a positive understanding about this disease; and (d) closure phase: the group members shared their feelings and experiences in this group, and a summary of each individual was made. The interventions were conducted by qualified psychological counselors who had at least 5 years of work experience in the psychotherapy. All of them had obtained the license of “National Consultant Psychologist Grade 2” and had received uniform training before they were involved in the interventions.

#### Evaluation of Therapeutic Efficacy

##### Changes in the Relevant Physiological and Psychological Indicators After 4, 8, and 12 Months of Treatment

The psychological indicators included: (1) Toronto Alexithymia scale (TAS). The TAS is a 20-item instrument that reflects the personality traits by measuring difficulty in identifying feelings, difficulty in describing feelings, illusion, and externally oriented thinking. A high TAS score means more severe alexithymia. (2) Hamilton Anxiety Scale (HAMA) [6] will be divided into 14 items, including 7 psychic anxiety factors, and 7 somatic anxiety factors. Each project is divided into 0–4 points (0 = no, 1 = mild, 2 = medium, 3 = severe, 4 = very severe), higher the score, higher the heavier patients’ anxiety level. (3), Hamilton Depression Scale (HAMD) [6] will be divided into 24 items, and each project is divided into 0–4 points (0 = no, 1 = mild, 2 = medium, 3 = severe, 4 = very severe), higher the score, higher the heavier patients’ depression level. (4) Ways of Coping Questionnaire (WCQ) [7] is a 20-item questionnaire and can be divided into positive coping subscales and negative coping subscales. Higher scores of the negative coping subscales mean more severe psychological problems, whereas higher scores of the positive coping subscales mean milder psychological problems. In our current study, the negative coping subscales were used. A senior psychiatric physician who was blind to the grouping scheme completed the evaluations using these tools. The physiological indicators including estradiol, prolactin, and progesterone were determined by ELISA using the reagent kits (Bioekon, Beijing, China). Venous blood samples were collected from the elbow during the luteal phase, and the tests were performed in strict accordance with the manufacturer’s instructions. A laboratory technician who was blind to the study groupings completed the tests independently.

Upon the completion of treatment, the efficacy in each group was evaluated based on the Criteria for the Diagnosis, Syndrome Differentiation, and Efficacy Evaluation of Hyperplastic Breast Diseases, which was established by the Professional Committee of Breast Surgery, China Association of Traditional Chinese Medicine [8]. The efficacy standards were as follows: (1) cured: the pain and mass were disappeared; (2) markedly improved: the maximal diameter of the mass was decreased by at least 1/4 and the pain disappeared; (3) improved: the maximal diameter of the mass was decreased by less than 1/4 and the pain was alleviated, or, the maximal diameter of the mass was decreased by more than 1/4 but the pain was not obviously alleviated; and (4) Non-effective: the mass showed no change or even becomes enlarged/stiffened, or only the pain is alleviated. The overall response rate is the sum of rates of “cured”, “markedly improved”, and “improved”. The patients were followed for one year via telephone or outpatients visits. The “relapse” was defined as the re-occurrence or even aggravation of the symptoms in a patient who had achieved at least “improved” and with her disease being stable for at least 6 months.

### Statistical Analysis

Statistical analysis was performed using the SPSS 17.0 software. Measurement data are presented as(±*s*). Since the values of physiological and psychological indicators at different time points were not independent in each group, repeated measures analysis of variance (rANOVA) was applied. The efficacies and relapse rates were compared using Chi square test. A value of *P* < 0.05 was considered statistically significant.

## Results

### Comparison of TAS Scores Before and After the Initiation of Treatment

The scores of TAS, HAMA, HAMD, WCQ negative coping factor subscales showed significant differences in various groups and at different time points (*P* < 0.05), and correlations were found among these factors (*P* < 0.05) (Table 2). Before the treatment, the scores of TAS, HAMA, HAMD, WCQ negative coping factor subscales were not significantly different among these three groups (*P* > 0.05). After 4, 8, and 12 months of treatment, the scores of TAS and WCQ negative coping subscales in the control group showed no significant differences when compared with the pre-treatment scores, while the HAMA and HAMD scores significantly decreased. In the AADM and PI groups, the scores of TAS, HAMA, HAMD, and WCQ negative coping subscales showed a declining trend after treatment. The scores of HAMA and HAMD in the 4th, 8th, and 12th month in the AADM group were significantly lower than those in the PI and control groups (*P* < 0.05), whereas the scores of TAS and WCQ negative coping subscales in the AADM group were significantly higher than the PI group and lower than the control group (*P* < 0.05). The PI group had significantly lower scores for HAMA and HAMD in the 4th, 8th, and 12th month than the control group (*P* < 0.05) (Table 3).

**Table 2:** Analysis of variance for the scores of TAS, HAMA, HAMD, and WCQ negative coping subscales before and after the treatment in three groups

Sources of variance	TAS		HAMA		HAMD		WCQ negative coping subscales	
	*F* value	*P* value	*F* value	*P* value	*F* value	*P* value	*F* value	*P* value
Grouping	3.10	0.0092	4.13	0.0032	3.67	0.0075	2.78	0.0192
Time	2.91	0.0121	3.25	0.0083	3.56	0.0083	2.86	0.0173
Grouping × time	2.96	0.0101	5.26	0.0011	3.16	0.0091	2.25	0.0236

**Table 3:** Comparison of variance of TAS, HAMA, HAMD, scores and WCQ negative coping subscales before and after treatment in three groups (*x* ± *s*)

Group	*N*	TAS	HAMA	HAMD	WCQ negative coping subscales
PI group	40				
Before treatment		78.78 ± 21.61	19.31 ± 5.11	22.90 ± 6.31	20.23 ± 5.73
Four months after treatment		73.12 ± 30.71^abc^	16.76 ± 7.21^abc^	18.06 ± 9.12^abc^	17.27 ± 10.65^abc^
Eight months after treatment		68.95 ± 22.36^abc^	12.43 ± 6.52^abc^	16.12 ± 8.54^abc^	14.82 ± 6.13^abc^
Twelve months after treatment		62.51 ± 19.25^abc^	10.62 ± 3.14^abc^	13.36 ± 8.67^abc^	12.09 ± 4.26^abc^
AADM group	40				
Before treatment		79.61 ± 22.34	19.36 ± 6.63	23.78 ± 7.06	19.27 ± 6.11
Four months after treatment		76.28 ± 23.23^ab^	14.60 ± 5.22^ab^	17.13 ± 6.07^ab^	18.25 ± 7.32^ab^
Eight months after treatment		74.23 ± 21.62^ab^	11.85 ± 4.27^ab^	14.27 ± 5.12^ab^	16.78 ± 6.57^ab^
Twelve months after treatment		72.03 ± 18.22^ab^	9.08 ± 4.65^ab^	11.63 ± 5.33^ab^	15.31 ± 4.26^ab^
Control group	40				
Before treatment		78.62 ± 22.01	18.26 ± 7.87	22.15 ± 6.91	21.38 ± 6.65
Four months after treatment		77.12 ± 22.83	16.35 ± 7.69^a^	20.25 ± 11.83^a^	20.71 ± 7.81
Eight months after treatment		77.34 ± 23.61	15.05 ± 6.42^a^	19.56 ± 10.69^a^	21.21 ± 5.33
Twelve months after treatment		76.68 ± 25.70	14.88 ± 6.57^a^	18.18 ± 9.57^a^	21.36 ± 5.09

^a^ *P* < 0.05, compared to the previous time points

^b^ *P* < 0.05, compared with the control group at the indicated time points

^c^ *P* < 0.05, compared with the AADM group at the same time points

### Comparison of the Estradiol, Prolactin, and Progesterone Levels Before and After Treatment Among Three Groups

The estradiol, prolactin, and progesterone levels showed significant differences in these groups and at different time points (*P* < 0.05), and correlations were observed among these factors (*P* < 0.05) (Table 4). The estradiol, prolactin, and progesterone levels were not significantly different before treatment among these three groups (*P* > 0.05). Whereas after the treatment, the estradiol and prolactin levels showed a declining trend but progesterone showed a rising trend in all three groups. The PI group had significantly lower estradiol and prolactin levels in the 4th, 8th, and 12th month than those in the control and AADM groups (*P* < 0.05)., while the progesterone levels in the 4th, 8th, and 12th month were significantly higher in the PI group compared to other two groups (*P* < 0.05). The AADM group had significantly lower estradiol and prolactin levels in the 4th, 8th, and 12th month than the control group (*P* < 0.05), whereas the progesterone levels in the 4th, 8th, and 12th month were significantly higher in the AADM group than the control group (*P* < 0.05) (Table 5).

**Table 4:** Analysis of variance of the estradiol, prolactin, and progesterone levels before and after treatment among three groups

Sources of variance	Estradiol		Prolactin		Progesterone	
	*F* value	*P* value	*F* value	*P* value	*F* value	*P* value
Group	2.85	0.0173	2.56	0.0282	2.91	0.0097
Time	4.56	0.0020	3.63	0.0087	2.51	0.0261
Group × time	2.76	0.0213	2.33	0.0310	2.00	0.0315

**Table 5:** Comparison of the estradiol, prolactin, and progesterone levels before and after treatment among three groups (*x* ± *s*)

Group	*N*	Estradiol (pmol/L)	Prolactin (ng/mL)	Progesterone (μg/L)
PI group	40			
Before treatment		608.32 ± 70.61	32.65 ± 7.11	8.82 ± 4.52
Four months after treatment		546.25 ± 91.21^abc^	28.10 ± 10.09^abc^	11.62 ± 5.66^abc^
Eight months after treatment		503.82 ± 72.52^abc^	24.77 ± 8.41^abc^	13.37 ± 6.07^abc^
Twelve months after treatment		487.71 ± 65.52^abc^	19.81 ± 7.31^abc^	14.05 ± 5.02abc
AADM group	40			
Before treatment		613.54 ± 69.56	32.73 ± 6.12	8.50 ± 4.27
Four months after treatment		580.20 ± 65.01^ab^	29.22 ± 6.62^ab^	10.01 ± 5.29^ab^
Eight months after treatment		563.32 ± 63.11^ab^	26.27 ± 7.02^ab^	12.65 ± 6.11^ab^
Twelve months after treatment		542.06 ± 60.07^ab^	23.60 ± 6.51^ab^	13.61 ± 6.45^ab^
Control group	40			
Before treatment		603.37 ± 71.13	31.98 ± 7.25	8.62 ± 6.45
Four months after treatment		591.56 ± 70.56^a^	30.24 ± 8.60^a^	9.57 ± 6.32^a^
Eight months after treatment		577.21 ± 68.15^a^	28.56 ± 7.59^a^	10.35 ± 5.16^a^
Twelve months after treatment		561.36 ± 68.75^a^	27.02 ± 6.33^a^	11.71 ± 5.32^a^

^a^ *P* < 0.05, compared with the previous time points

^b^ *P* < 0.05, compared with the control group at the same time point

^c^ *P* < 0.05, compared with the AADM group at the same time point

### Comparison of the Efficacy and the Relapse Rate During the 1-Year Follow-Up Among Three Groups

The overall response rate was significantly lower in the control group (65 %) than the AADM group (85 %) ((χ^2^ = 4.267, *P* < 0.05) and PI group (90 %) (χ^2^ = 7.168, *P* < 0.05). However, it was not significantly different between AADM group and PI group (χ^2^ = 0.457, *P* > 0.05) (Table 6). The relapse rate was 69.23 % in the control group, 61.76 % in the AADM group, and 25 % in the PI group. Thus, the relapse rate was significantly lower in PI group than the control group (χ^2^ = 12.014, *P* < 0.05) and compared to AADM group (χ^2^ = 9.651, *P* < 0.05); however, it was not significantly different for both control and AADM groups (χ^2^ = 0.361, *P* > 0.05).

**Table 6:** Comparison of clinical efficacies among three groups (*n*, %)

Group	*N*	Cured	Markedly improved	Improved	Non-effective	Overall response rate
Control group	40	6 (15)	7 (17.5)	13 (32.5)	14 (35)	26 (65)
AADM group	40	11 (27.5)	16 (40)	7 (17.5)	6 (15)	34 (85)
PI group	40	25 (62.5)	6 (15)	5 (12.5)	4 (10)	36 (90)

## Discussion

BH can be caused by a variety of factors, among which the psychological factors play a key role in the occurrence and development of BH [4, 9]. Research has shown that most patients with BH have adverse psychological responses such as anxiety and depression. The degree of psychological stress reaction is determined by two aspects: the degree of the individual’s intrinsic and extrinsic stress reaction. Notably, the personality traits determine the degree of the individual’s intrinsic stress reaction, whereas some external factors such as personal experiences determine the degree of the individual’s extrinsic stress reaction. Research has shown that the personality traits of patients with BH tend to be neurotic and introverted, resulting in higher degree of the individual’s intrinsic stress reaction. In addition, these patients have higher incidence of adverse life events and are often under heavier pressure from work and life, which increases their degree of extrinsic stress reaction [10, 11]. Since both the intrinsic and extrinsic stresses are higher among these patients, they are prone to strong stress reactions, which are manifested as adverse psychological reactions such as depression and anxiety [10, 11]. As a negative life event, the BH itself can trigger the extrinsic stress reaction and further exacerbate the negative emotions, forming a vicious circle. Once the adverse stress reactions occur, they can interfere with the normal functions of the hypothalamus–pituitary–adrenal axis, leading to the abnormal secretion of estrogen and progesterone. The abnormally high estrogen stimulates the hyperplasia of mammary gland. Meanwhile, the decreased secretion of progesterone and the increased prolactin level further suppress the progesterone level, which decreases the progesterone/estradiol ratio and further enhances the stimulation of estrogen on breast tissue. Finally, the normal cyclical proliferation + involution of the breast becomes disordered, resulting in mammary hyperplasia [11, 12].

Currently, the mainstream treatment for this condition is use of estrogen receptor antagonist to fight against the excessively secreted estrogen. However, the estrogen receptor antagonist cannot essentially restore the already disordered endocrine system, and therefore the relapse rate remains high [13]. PI can effectively reduce the patient’s adverse stress reactions and thus basically restore the endocrine system and these interventions are easily accepted by the patients. However, few systematic studies have explored the role of PI in patients with BH. Most psychotherapies are based on a single treatment mode and last for a short period (within half a year), and the relapse rates are high. In our current study, a full-course psychological intervention with long follow-up was performed, with an attempt to determine the efficacy of PI. Integrated psychotherapy was adopted in our study. It had following advantages: (1) Cognitive-behavioral therapy: Cognitively, it corrects the patients’ negative perceptions (incorrect knowledge and excessive fear) on the BH; behaviorally, it timely improves the existing bad behaviors that may exacerbate the disease, thus alleviating the disease conditions and relieving the anxiety. (2) Structured closed group therapies: Patients in the group suffer from the same disease, when asked to meet together, they can communicate deeply about their diseases, become empathized, and establish a sense of belonging, which is helpful to remove the psychological resistance/defense of BH patients with introverted characteristics, expose their personality mode, and thus facilitate the guided therapy performed by psychotherapists. (3) Family therapy: A survey on the psychological status of BH patients has shown that, among the adverse life events, family events are more aggravating than work, learning, social activities, and other issues [14]. Thus, adverse family events are closely associated with the poor psychological reactions in BH patients. In our current study, the family therapy enabled the patients to obtain the maximal support from their family members; the patients could be better understood by their family members, and good familial relationships and smooth communication channels were established. All these were particularly helpful to reduce the degree of extrinsic stress reactions. Thus, our study demonstrated that the personality traits and coping ways (and thus the psychological status and endocrine disorders) could be better adjusted in the PI group than the AADM and control groups in terms of effectiveness, duration, and relapse. The PI group also had superior overall response rate than the control and AADM groups. Although the AADM group was more effective than the PI group in alleviating the anxiety and depression and had the similar short-term therapeutic effectiveness, the relapse rate was higher after drug withdrawal and therefore the long-term efficacy was poor.

Our study was limited by its small sample size and single-center design. Multi-center coordinated studies with larger sample sizes are required to yield more accurate findings. Currently, to our knowledge no early identification and warning system for the risk factors of the psychological problems among BH patients is available. No efficacy evaluation indicator system or criteria has been established for the PI for the common psychological problems among BH patients. Finally, no study has been performed on the selection, application, and promotion of the optimized PI. In future, we will further compare and evaluate the effectiveness of different PI on BH, with an attempt to develop an early identification and warning system for the risk factors of the psychological problems among these patients, establish efficacy evaluation indicator system or criteria for the PI. And finally develop the optimized PI and the clinical pathways of psychosomatic treatment.
